# Leucyl-tRNA synthetase deficiency systemically induces excessive autophagy in zebrafish

**DOI:** 10.1038/s41598-021-87879-4

**Published:** 2021-04-16

**Authors:** Masanori Inoue, Hiroaki Miyahara, Hiroshi Shiraishi, Nobuyuki Shimizu, Mika Tsumori, Kyoko Kiyota, Miwako Maeda, Ryohei Umeda, Tohru Ishitani, Reiko Hanada, Kenji Ihara, Toshikatsu Hanada

**Affiliations:** 1 Department of Cell Biology, Oita University Faculty of Medicine, Yufu, Oita 879-5593 Japan; 2 Department of Pediatrics, Oita University Faculty of Medicine, Yufu, Oita 879-5593 Japan; 3 Department of Neuropathology, Institute for Medical Science of Aging, Aichi Medical University, Aichi, 480-1195 Japan; 4 Department of Neurophysiology, Oita University Faculty of Medicine, Yufu, Oita 879-5593 Japan; 5 Division of Cellular and Molecular Biology, Department of Homeostatic Regulation, Research Institute for Microbial Diseases, Osaka University, Osaka, 565-0871 Japan

**Keywords:** Diseases, Molecular medicine, Pathogenesis

## Abstract

Leucyl-tRNA synthetase (LARS) is an enzyme that catalyses the ligation of leucine with leucine tRNA. LARS is also essential to sensitize the intracellular leucine concentration to the mammalian target of rapamycin complex 1 (mTORC1) activation. Biallelic mutation in the *LARS* gene causes infantile liver failure syndrome type 1 (ILFS1), which is characterized by acute liver failure, anaemia, and neurological disorders, including microcephaly and seizures. However, the molecular mechanism underlying ILFS1 under LARS deficiency has been elusive. Here, we generated Lars deficient (*larsb*^*−/−*^) zebrafish that showed progressive liver failure and anaemia, resulting in early lethality within 12 days post fertilization. The *atg5*-morpholino knockdown and bafilomycin treatment partially improved the size of the liver and survival rate in *larsb*^*−/−*^ zebrafish. These findings indicate the involvement of autophagy in the pathogenesis of *larsb*^*−/−*^ zebrafish. Indeed, excessive autophagy activation was observed in *larsb*^*−/−*^ zebrafish. Therefore, our data clarify a mechanistic link between LARS and autophagy in vivo. Furthermore, autophagy regulation by LARS could lead to development of new therapeutics for IFLS1.

## Introduction

Aminoacyl-tRNA synthetases (ARSs) are essential enzymes that catalyse the ligation of amino acids to their cognate transfer RNAs (tRNAs), which is the first step in protein synthesis^1–4^. Leucyl-tRNA synthetase (LARS), a component of the multi-tRNA synthetase complex, is critical for charging leucine tRNA with leucine^3^. Furthermore, LARS has a non-canonical role as a mammalian target of rapamycin complex 1 (mTORC1)-associated protein required for amino acid-induced mTORC1 activation, indicating that LARS is not only a tRNA synthetase, but also an intracellular leucine sensor for mTORC1 signalling^5–8^.

The alternative functions of ARSs play a critical role in cellular homeostasis, including translation control, transcription regulation, cell migration, inflammatory responses, tumorigenesis, and cell death regulation^9,10^. These functions may explain the mechanisms of several human diseases caused by ARS gene mutations, including cancer, neurological disorders, and autoimmune diseases^4,11–14^. Biallelic mutation in the cytoplasmic *LARS* leads to an infantile hepatopathy called infantile liver failure syndrome type 1 (ILFS1), which is characterized by acute liver failure in the first few months and is associated with failure to thrive, anaemia, microcephaly, muscular hypotonia, and seizures^15,16^.

Although LARS is involved in mTORC1 pathways and its dysfunction may be responsible for ILFS1 pathology, the function of LARS in vivo has remained elusive. Previous research using a Lars loss of function (*larsb*^*−/−*^) zebrafish model revealed that the mutant zebrafish exhibit a phenotype similar to that of ILFS1^17^. Moreover, in contrast to a previous study showing that ablation of LARS desensitizes the mTORC1 pathway to amino acids in yeast and human cell lines^5,6^, the *larsb*^*−/−*^ zebrafish shows augmented mTORC1 activation^17^. Furthermore, suppression of mTORC1 activation by rapamycin treatment or knockdown of mTORC1 by morpholino partially rescues the phenotype of *larsb*^*−/−*^ zebrafish^17^.

Therefore, to gain further insight into the LARS-mTORC1-autophagy circuit, we examined the involvement of autophagy in the pathogenesis of *larsb*^*−/−*^ zebrafish.

## Results

### Generation of *larsb*^−/−^ zebrafish

To assess the function of LARS in vivo, we generated larsb-knockout (*larsb*^***−/−***^) zebrafish using CRISPR/Cas9 technology^18,19^. Two genes, *larsa* and *larsb* encode cytosolic Lars in zebrafish, and among them, *larsb* shares higher homology with human *LARS*.

**Figure 1:**
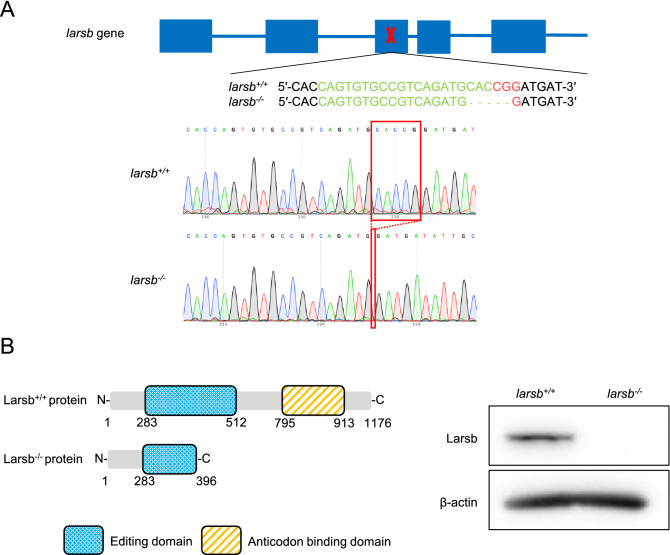
Construction of *larsb*-knockout mutant zebrafish line. **(A)** Diagram showing the *larsb* genomic locus, CRISPR/Cas9 target site, and *larsb*-knockout (*larsb*^*−/−*^) zebrafish mutant genotype. The sgRNA target sequence is displayed in green and the PAM region in red. In the genomic sequencing analysis chromatograms, the deletion region in the mutant *larsb*^*−/−*^ zebrafish is shown by the red box. **(B)** The Lars protein of *larsb*^*−/−*^ zebrafish had a missing editing domain. Western blot analysis of the Larsb protein expression in *larsb*^+*/*+^ and *larsb*^*−/−*^ zebrafish. β-actin levels served as the loading control. Larsb: leucyl-tRNA synthetase b.

To obtain *larsb* mutant zebrafish, we designed the CRISPR/Cas9 target site in exon 3 of *larsb* (Fig. 1A), which corresponds to the editing domain of the Lars protein (Fig. 1B). Notably, most *LARS* gene mutations in humans occur in the editing domain^15,16,20^, indicating that this domain has an essential function in vivo. After screening several founders that transmitted targeted indels to the F1 progeny, we established a stable line with a frameshift mutation caused by a 5-bp deletion (Fig. 1A). Western blotting confirmed a complete lack of the Lars protein in *larsb*^*−/−*^ larvae (Fig. 1B). Furthermore, we performed a quantitative PCR assay to analyze the mRNA levels encoding the proteins responsible for the canonical function of Leucyl-tRNA synthetase, Larsa, Larsb, and Lars2 (Supplementary Fig S1). There was almost no expression of larsa mRNA when compared with that of larsb, indicating that *larsa* may be a pseudogene. Meanwhile, the mRNA expression of Lars2, a mitochondrial leucyl-tRNA synthetase that can charge mitochondrial tRNA with its cognate amino acids, significantly increased in *larsb*^*−/−*^ zebrafish. It may be that lars2 expression is induced by the *larsb* gene knockout via a molecular mechanism, such as nonsense-induced transcriptional compensation (NITC)^21,22^, resulting in the relatively mild phenotype in *larsb*^*−/−*^ zebrafish.

### Liver defects and early lethality in *larsb*^−/−^ zebrafish

**Figure 2:**
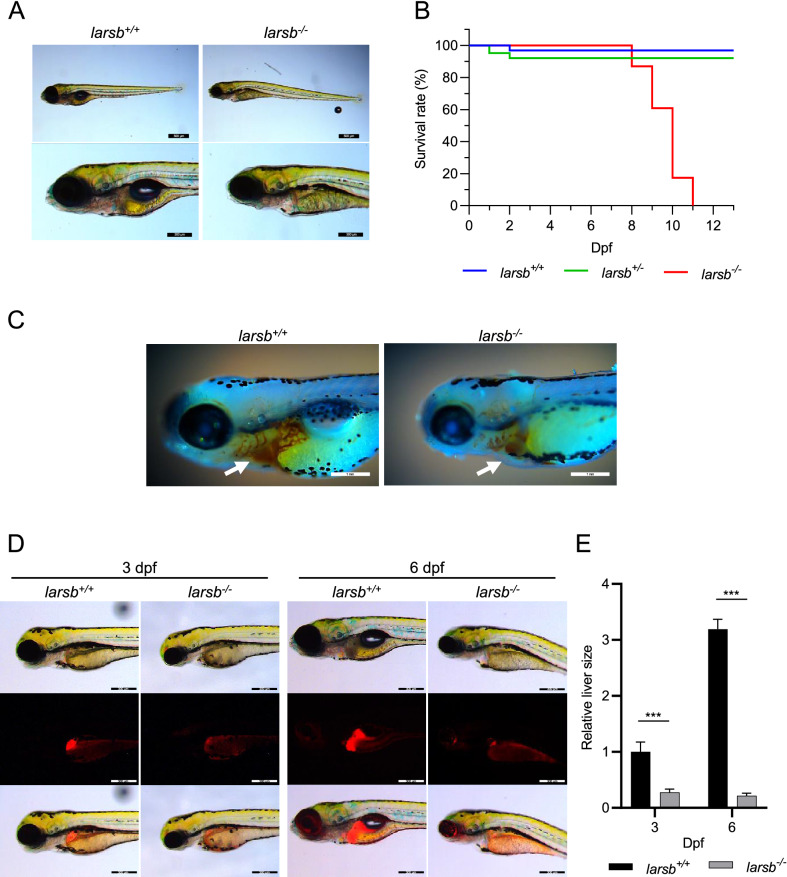
*Larsb*-knockout larvae display severe developmental phenotype and liver abnormality with early lethality. **(A)** Bright field lateral views of *larsb*^+*/*+^ and *larsb*^*−/−*^ embryos at 96 h post fertilization (hpf). Scale bar: 500 μm (top row) and 300 μm (bottom row). **(B)** Kaplan–Meier survival curve of *larsb*^+*/*+^ (n = 32), *larsb*^+*/-*^ (n = 63), and *larsb*^*−/−*^ (n = 23) larvae. **(C)** Lateral views of *larsb*^+*/*+^ and *larsb*^*−/−*^ embryos containing haemoglobin-containing cells (white arrows) stained with o-dianisidine at 72 hpf. **(D)** Morphological abnormality at 3 dpf and 6 dpf in the livers of *larsb*^*−/−*^ larvae under Tg[*fabp10*:mcherry] background. Scale bar: 300 μm. **(E)** Quantification of liver size in larsb^−/−^ larvae under Tg[*fabp10*:mcherry] background (3 dpf and 6 dpf). Liver sizes were evaluated using ImageJ software version 1.52a (https://imagej.nih.gov/ij/). n = 5 fish/group. Error bars indicate SEM. Student’s t-test; ***P < 0.001. Statistics were calculated and the figure was produced in GraphPad software version 8 (https://www.graphpad.com/scientific-software/prism/). Larsb: leucyl-tRNA synthetase b, dpf: days post fertilization.

*Larsb *^*−/−*^ larvae had hatching rates and timings comparable with that of *larsb*^+*/*+^ larvae. However, all *larsb*^*−/−*^ larvae exhibited thinness, cardiac edema, and swim bladder deflation (Fig. 2A). All *larsb*^*−/−*^ larvae died between 8 and 11 days post fertilization (dpf) (Fig. 2B). Because anaemia is one of the typical symptoms in ILFS1 patients, we performed o-dianisidine staining to detect haemoglobin-containing cells in *larsb*^*−/−*^ larvae. As expected, the *larsb*^*−/−*^ larvae showed anaemia (Fig. 2C)^15^.

We further analysed the morphological changes in liver development by crossing *larsb*^*−/−*^ zebrafish with Tg[*fabp10:*mcherry] transgenic zebrafish, which express mCherry fluorescent protein specifically in the liver^23,24^. The livers of *larsb*^*−/−*^ larvae were significantly smaller than that of *larsb*^+*/*+^ larvae at 3 dpf, and showed no further development until their death (P < 0.001; Fig. 2D,E). These data indicate the similarity of *larsb*^*−/−*^ zebrafish phenotype to ILFS1 due to human *LARS* mutation. Thus, the in vivo function of LARS seems to be conserved across zebrafish and humans.

As previously described, the primary symptoms of mutations in *GARS*, *SARS*, *HARS*, and other tRNA-synthetase genes are neurological defects^4,25^. The *larsb*^*−/−*^ zebrafish also showed microcephaly and loss of locomotor activity (Supplementary Figs. S2A–F). These data indicate that larsb could have an essential role in the development of the neuronal system, as previously described in other tRNA synthetases^4,25^.

### Lars deficiency induces autophagy in *larsb*^−/−^ zebrafish

**Figure 3:**
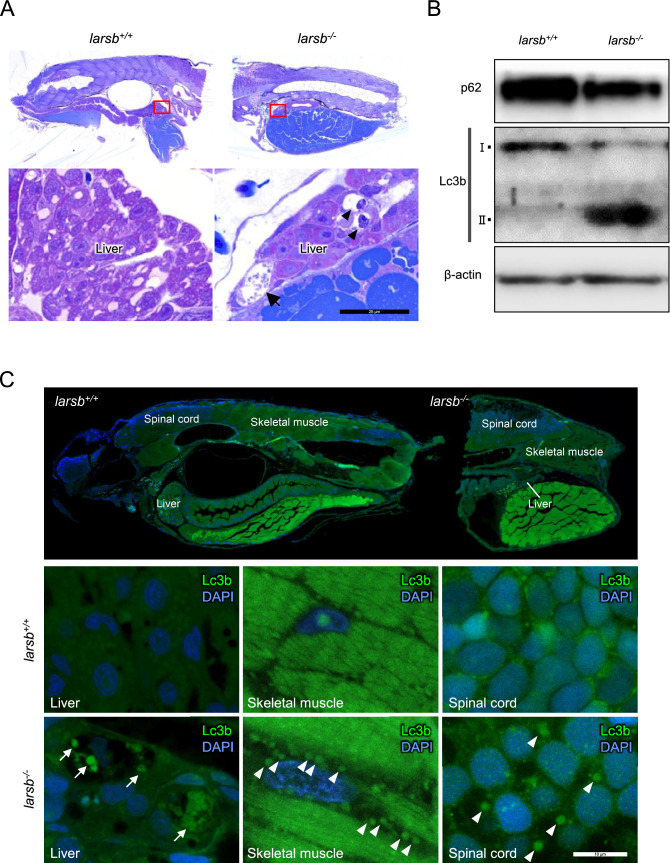
Histopathology and fluorescent immunostaining of *larsb*-knockout larvae. **(A)** Lower magnification sagittal views (top row) and higher magnification views (bottom row) of *larsb*^+*/*+^ and *larsb*^*−/−*^ larvae. Huge vacuolations, which seemed to disappear in the cytoplasm, were seen in the livers of *larsb*^*−/−*^ larvae (black arrows), and some large vacuolations included a bare nucleus (black arrowheads). Scale bar: 25 µm. **(B)** Western blot analysis of p62 and Lc3b protein expression in *larsb*^+*/*+^ and *larsb*^*−/−*^ larvae. β-actin levels served as the loading control. **(C)** Lower magnification sagittal views of *larsb*^+*/*+^ and *larsb*^*−/−*^ larvae (top row). Fluorescent immunostaining against Lc3b (green) and DAPI (blue) of the livers, skeletal muscles, and spinal cords of *larsb*^+*/*+^ and *larsb*^*−/−*^ larvae. Livers of *larsb*^*−/−*^ larvae had large vacuoles, including floating nuclei and various sized dots with Lc3b immunoreactivity (white arrows). Skeletal muscles and spinal cords of *larsb*^*−/−*^ larvae had many dots with Lc3b immunoreactivity (white arrowheads). Scale bar: 10 µm. Lars: leucyl-tRNA synthetase; Lc3b: microtubule-associated protein 1A/1B-light chain 3; DAPI: 4′,6-diamidino-2-phenylindole.

To assess liver abnormalities in *larsb*^*−/−*^ zebrafish, we performed histopathological examination. The livers of *larsb*^*−/−*^ larvae drastically reduced in size compared with that of *larsb*^+*/*+^ larvae (Fig. 3A). In addition, large vacuolations, which seemed to disappear in the cytoplasm, were observed in the livers of *larsb*^*−/−*^ larvae. Some large vacuolations included a bare nucleus. These findings indicate autophagic cell death^26^. Indeed, microtubule-associated protein 1A/1B-light chain 3 (LC3B)-II, a standard marker of autophagosome formation, was upregulated in *larsb*^*−/−*^ larvae (Fig. 3B), as shown by western blotting. The selective autophagy substrate p62 was also more degraded in *larsb*^*−/−*^ larvae than in *larsb*^+*/*+^ larvae (Fig. 3B). However, histologically, cytoplasmic condensation, cytoplasmic blebbing, and fragmented nuclei, which indicate apoptotic cell death, were not observed in the livers of *larsb*^*−/−*^ larvae. These results indicate that apoptotic cell death is not induced by Lars deficiency.

Next, to examine whether autophagy is involved in liver abnormalities, we evaluated the status of autophagy by fluorescent immunostaining for Lc3b in *larsb*^*−/−*^ larvae under Tg[*fabp10*:mcherry] background. Lc3b, a downstream constituent of the autophagy pathway and participant in autophagosome formation, is widely used to monitor autophagy^27^. Although *larsb*^+*/*+^ larvae had no apparent autophagic structures in the liver, *larsb*^*−/−*^ larvae displayed large vacuoles, including floating nuclei and various sized dots with Lc3b immunoreactivity, thereby indicating autophagic cell death (Fig. 3C). Hepatocellular nucleophagy, showing fragmented nuclei labelled with Lc3b, was also observed in the livers of *larsb*^*−/−*^ larvae. Moreover, many autophagosomal structures visualized with Lc3b were also observed in the skeletal muscles and spinal cords of *larsb*^*−/−*^ larvae in comparison to *larsb*^+*/*+^ larvae. Thus, these data indicate that Lars deficiency induces autophagy not only in the liver, but also in the central nervous system and skeletal muscle during the early embryonic stage.

### Immunoelectron microscopy analysis of *larsb*^−/−^ larvae under Tg[fabp10:mcherry] background

**Figure 4:**
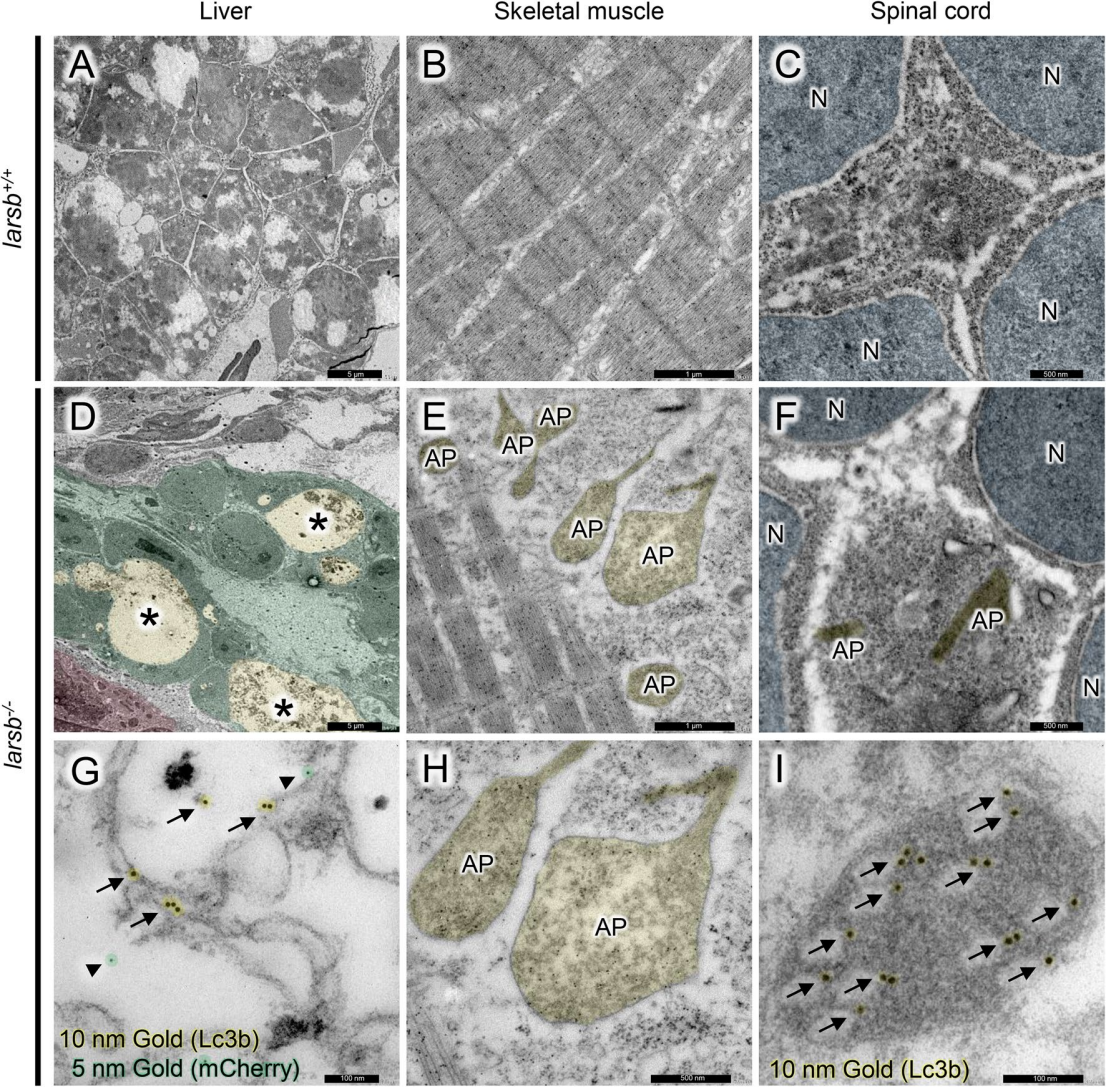
Immunoelectron microscopy of *larsb-*knockout larvae under Tg[*fabp10*:mcherry] background. **(A–C)** Immunoelectron microscopy of the liver, skeletal muscle, and spinal cord of *larsb*^+*/*+^ larva. **(D–I)** Immunoelectron microscopy of the liver, skeletal muscle, and spinal cord of *larsb*^*−/−*^ larva. The bottom row shows higher magnification images **(G–I)**. Large vacuoles in the livers of *larsb*^*−/−*^ larvae (asterisks) were composed of numerous irregular membranous structures, which showed immunoreactivity against both Lc3b (black arrows) and mCherry (black arrowheads) **(D,G)**. Scale bar: 5.0 µm for **(A,D)**; 1.0 µm for B and E; 500 nm for **(C,F,H)**; 100 nm for **(G,I)**. AP: autophagosome, N: nucleus, *Larsb:* leucyl-tRNA synthetase, Lc3b: microtubule-associated protein 1A/1B-light chain 3.

We next assessed the ultrastructure of the liver, skeletal muscle, and spinal cord by immunoelectron microscopy. There were no overt autophagic structures in the livers, skeletal muscles, and spinal cords of *larsb*^+*/*+^ larvae (Figs. 4A–C). However, large vacuoles in the livers of *larsb*^*−/−*^ larvae were composed of numerous irregular membranous structures with immunoreactivity against both Lc3b and mCherry (Fig. 4D,G). The mCherry protein was detected using a red fluorescent protein (RFP) antibody to confirm that the Lc3b-positive cells were hepatocytes. Many irregular structures labelled with anti-Lc3b antibody, which were presumed to be autophagosomes or autolysosomes, were also observed in the muscles and spinal cords of *larsb*^*−/−*^ larvae compared with those of *larsb*^+*/*+^ larvae (Fig. 4E,F,H,I). Therefore, although autophagy caused by Larsb deficiency occurred in some tissues, including the skeletal muscle and spinal cord, the liver was the most damaged tissue in *larsb*^*−/−*^ zebrafish.

### Inhibition of autophagy partially rescues the liver defects

To verify whether the liver defects and severe developmental abnormalities in *larsb*^*−/−*^ larvae were due to autophagy, we performed a knockdown experiment using an antisense morpholino for *atg5* (atg5-MO), which is essential for autophagy induction^28^. As a highly efficient *atg5* knockdown in zebrafish causes abnormal neuronal development^29^, the amount of MO injected was estimated to achieve a knockdown efficiency of 60% (Supplementary Figs. S3A and B).

**Figure 5:**
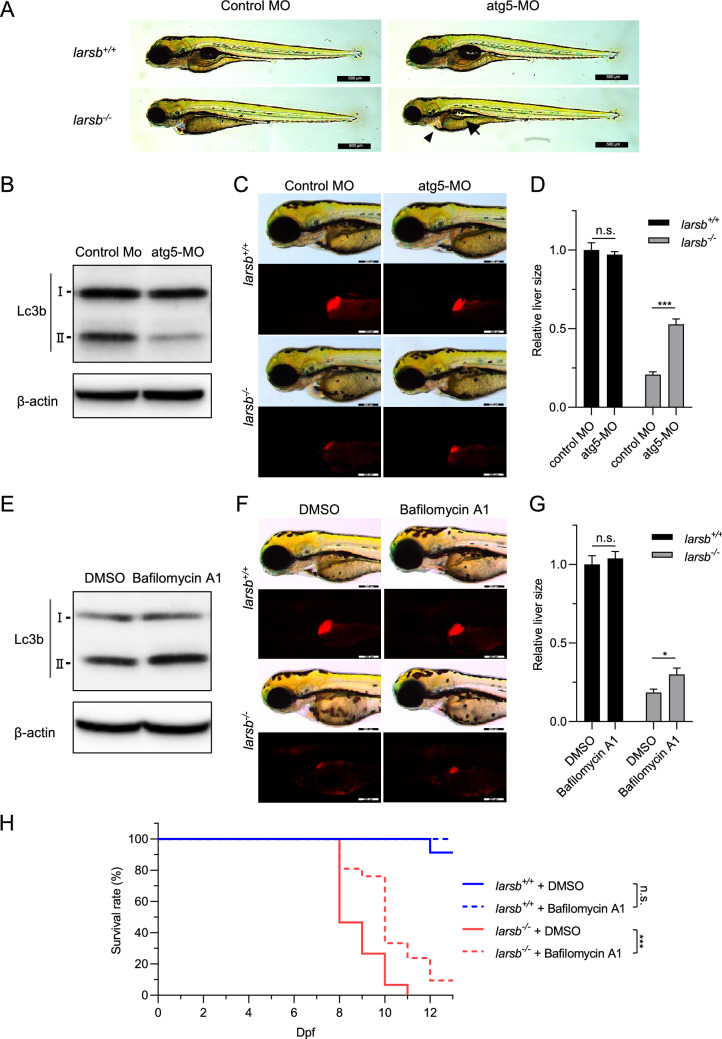
Inhibition of autophagy prevents abnormal development and improves survival in *larsb-*knockout larvae. **(A)** Morphology of *larsb*^+*/*+^ and *larsb*^*−/−*^ embryos injected with either control MO or atg5-MO (72 h post fertilization (hpf)). Scale bars: 500 µm. **(B)** Western blot analysis of Lc3b protein expression at 72 hpf for wild-type embryos injected with either control MO or atg5-MO. β-actin levels served as the loading control. **(C)** Morphological abnormality at 72 hpf in the livers of *larsb*^*−/−*^ larvae under Tg[*fabp10*:mcherry] background injected with either control MO or atg5-MO. Scale bars: 200 μm. **(D)** Quantification of liver size in *larsb*^*−/−*^ larvae under Tg[*fabp10*:mcherry] background (72 hpf). Liver sizes were evaluated using ImageJ software version 1.52a (https://imagej.nih.gov/ij/). n = 4 fish/group. Error bars indicate SEM. Student’s t-test; ***P < 0.001. **(E)** Western blot analysis of Lc3b protein expression at 72 hpf for wild-type embryos treated with DMSO or bafilomycin A1. β-actin levels served as the loading control. **(F)** Morphological abnormality at 72 hpf in the livers of *larsb*^*−/−*^ larvae under Tg[*fabp10*:mcherry] background treated with DMSO or bafilomycin A1. Scale bars: 200 μm. **(G)** Quantification of liver size in *larsb*^*−/−*^ larvae under Tg[*fabp10*:mcherry] background (72 hpf). Liver sizes were evaluated using ImageJ software version 1.52a (https://imagej.nih.gov/ij/). n = 10 fish/group. Error bars indicate SEM. Student’s t-test; *P < 0.05. (H) Kaplan–Meier survival curve of *larsb*^+*/*+^ (n = 23) and *larsb*^*−/−*^ (n = 15) larvae treated with DMSO and *larsb*^+*/*+^ (n = 11) and *larsb*^*−/−*^ larvae (n = 21) treated with bafilomycin A1. Statistics were calculated and the figure was produced in GraphPad software version 8 (https://www.graphpad.com/scientific-software/prism/). *Larsb:* leucyl-tRNA synthetase b, MO: morpholino, n.s.: non-significant, DMSO: dimethyl sulfoxide, Dpf: days post fertilization.

As expected, atg5-MO prevented abnormal embryonic development, such as cardiac edema and swim-bladder deflation in *larsb*^*−/−*^ larvae (Fig. 5A). The effect of atg5-MO on autophagy was confirmed by western blot analysis for Lc3b-II (Fig. 5B). The atg5-MO reduced the conversion of Lc3b-I to Lc3b-II, indicating an effective inhibition of autophagy.

Atg5-MO also partially rescued the liver defects in *larsb*^*−/−*^ larvae ((Fig. 5C,D). However, atg5-MO did not improve the survival rate of *larsb*^*−/−*^ zebrafish, presumably because of its transient effectiveness for up to 5 days after injection (Supplementary Fig. S4).

To validate whether autophagy is involved in the *larsb*^*−/−*^ phenotype, we treated *larsb*^*−/−*^ larvae with the specific autophagy inhibitor bafilomycin A1. The effect of autophagy inhibition was estimated by western blot analysis for Lc3b-II. Bafilomycin A1 augments Lc3b-II accumulation because it inhibits autophagosomal fusion and degradation^30^. As expected, bafilomycin A1 treatment increased Lc3b-II accumulation, indicating that it effectively inhibits autophagy in zebrafish (Fig. 5E). Bafilomycin A1 treatment also partially improved the size of the liver in *larsb*^*−/−*^ larvae (Fig. 5F,G). Notably, we observed a substantial improvement in cardiac edema after treating *larsb*^*−/−*^ larvae with bafilomycin A1 as well as when *atg5* was knocked down (Fig. 5D). The survival rate was also significantly enhanced by bafilomycin A1 treatment (Fig. 5H).

To further validate the involvement of autophagy in the *larsb*^*−/−*^ phenotype, *larsb*^*−/−*^larvae were treated with the other autophagy inhibitors, chloroquine and 3-methyladenin. Both autophagy inhibitors effectively improved the liver phenotype of *larsb*^*−/−*^ larvae (Supplementary Figs. S5A and B). The survival rate was also significantly enhanced by chloroquine treatment (Supplementary Fig. S5C), but not 3-methyladenine, which may be linked to the high genotoxicity of 3-methyladenine^31^ (Fig. 5F).

In contrast, treatment with the mTORC1 inhibitor rapamycin had no effect on the survival of *larsb*^*−/−*^ larvae (Supplementary Fig. S6B) but retarded *growth as reported previously*^32^. This indicates that the larger liver observed in the rapamycin-treated *larsb*^*−/−*^ zebrafish may have been due to delayed liver phenotype progression, rather than rapamycin treatment (Supplementary Fig. S6A)*.*

These experiments provide direct evidence that hyperactivated autophagy induced by Lars deficiency is responsible for the liver defects and an early lethality.

## Discussion

In this study, we provide evidence on the in vivo function of LARS in autophagy regulation. *Larsb*^*−/−*^ zebrafish displayed liver failure and anaemia, a phenotype similar to ILFS1 caused by human *LARS* gene mutations. Histopathological analysis of *larsb*^*−/−*^ zebrafish showed enhanced autophagy not only in the liver, but also in other tissues, including the nervous system and muscles, during early embryonic development. In addition, huge vacuolations with bare nuclei were observed in the livers of *larsb*^*−/−*^ zebrafish, indicating severe autophagic cell death. Inactivation of autophagy by *atg5* knockdown or bafilomycin treatment partially rescued early lethality with liver failure. These results imply that the loss-of-function mutations of *LARS* in ILFS1 cause severe autophagic cell death in the liver.

Previously, in vitro studies have shown that LARS induces mTORC1 activation by sensing abundant intracellular leucine concentration, thereby inhibiting autophagy^5,6^. In contrast, LARS dysfunction activates autophagy by inhibiting mTORC1 activity^5,6^. These findings indicate the essential function of LARS in regulating autophagy. Wang et al. through in vivo studies have shown that *larsb*^*−/−*^ zebrafish have severe liver failure and increased mTORC1 activation^17^. Rapamycin, an mTORC1 inhibitor, partially rescues liver failure in *larsb*^*−/−*^ zebrafish, suggesting that hyperactivation of mTORC1 may be related to the onset of ILFS1^17^. Therefore, there seems to be a discrepancy between the in vitro and in vivo experiments.

Our histopathological data clearly showed that *larsb*^*−/−*^ zebrafish had increased autophagy in several tissues, including the skeletal muscle and central nervous system as well as the liver. Systemic autophagy induced by Lars deficiency could explain the general symptoms of ILFS1, such as muscle hypotonia, mental retardation, and convulsions^15,16^. Notably, Lars deficiency-induced autophagy caused significant damage to the liver. In the muscle tissue, the mTORC1-dependent autophagy pathway is mainly regulated by insulin signalling, whereas in the liver, it is strongly regulated by amino acid concentrations^33^. As LARS is a leucine concentration sensor for amino acid signalling to mTORC1, LARS may play an essential role in autophagy regulation, especially in the liver.

Our experiments suggest a mechanistic link between ILFS1 and LARS loss-of-function mutations. Although rapamycin did not affect the phenotype of *larsb*^*−/−*^ larvae, the atg5-morpholino, chloroquine, and the lysosome-targeting autophagy inhibitor bafilomycin A1, partially improved the survival rate and prevented liver damage. *Atg5* is an important autophagy gene that forms an Atg12-Atg5-Atg16 multimetric complex and plays an essential role in autophagosome membrane expansion and completion^34–36^. Morpholino knockdown of *atg5* has been reported to show successful inhibition of autophagy^37^. Our experiment also showed the efficient knockdown of Atg5 protein expression and the suppression of Lc3b-II conversion, indicating an efficient inhibition of autophagy in vivo.

Notably, the concentration of bafilomycin, an inhibitor of vacuolar H^+^ ATPase (V-ATPase), used in the rescue experiment was relatively low (2.5 nM). It has a variety of effects, not only in the inhibition of autophagy, but also the inhibition of cell growth and induction of apoptosis and differentiation^38^. To achieve the efficient inhibitory effects on autophagic degradation, bafilomycin A1 is usually required at high concentrations (> 100 nM). However, it also induces severe acidosis and secondary adverse effects in zebrafish larvae^39^. In fact, larvae died soon after treatment with bafilomycin A1 at 250 nM in our experiments. Although 25 nM of bafilomycin A1 improved the survival rate of *larsb*^*−/−*^ larvae, it did not rescue these liver defects. Therefore, we decided to conduct the rescue experiment with bafilomycin A1 at a concentration of 2.5 nM to prevent toxicity for growth on larvae. Importantly, 2.5 nM of bafilomycin A1 sufficiently accumulated Lc3b-II protein in larvae, suggesting that it effectively inhibits autophagy.

Our results suggested that suppression of excessive autophagy may rescue the symptoms of ILFS1. Of note, *larsb*^*−/−*^ zebrafish exhibited a more severe phenotype than ILFS1, although the phenotype closely resembled the symptoms of ILFS1. The exact molecular mechanism by which *LARS* mutation influences human ILFS1 needs to be determined using knock-in animal models, wherein a corresponding mutation is introduced into the zebrafish *larsb* locus. Moreover, there is increasing evidence for autophagy being associated with many diseases, including sepsis, Parkinson's disease, and Alzheimer's disease^40–42^. Hence, autophagy regulation by *LARS* may lead to new therapeutics for these related disorders.

## Methods

### Zebrafish maintenance

Zebrafish AB genetic background *larsb* mutant and Tg[*fabp10*:mcherry]^23,24^ were raised and maintained following standard procedures. They were kept at 28–29 °C under a 14-h:10-h light:dark cycle. Embryos were collected and housed at 28.5 °C. All animal experimental procedures were performed in accordance with the institutional and national guidelines and regulations. The study was carried out in compliance with the ARRIVE guidelines. The study protocol was approved by the Institutional Review Board of Oita University (approval no. 180506).

### Generation of the *larsb*^*−/−*^ zebrafish line

A *larsb*^*−/−*^ zebrafish line was generated via CRISPR/ Cas9 gene editing^18,19^. The site of the *larsb* sgRNA target was 5′-CAGTGTGCCGTCAGATGCACCGG-3′, in the editing domain of the LARS protein. Cas9 protein (300 pg) and gRNA (30 pg) were injected into one-cell-stage wild-type embryos. The mutation at the target site was verified via Sanger sequencing. The injected embryos were raised until adulthood and outcrossed with wild-type adults. DNA extracted from the F1 generation of whole larvae at 24 h post fertilization (hpf) was screened for indels by the heteroduplex mobility assay^43,44^ and Sanger sequencing. The F0 founder with germline transmission was selected to establish the knockout zebrafish line. F1 generations were raised to adulthood, had their fins clipped, and were sequenced. Fish carrying the same mutation (deletion of CACCG) were identified. All experiments were performed on embryos from the F2 or F3 progeny.

### Generation of transgenic zebrafish

Tg[*fabp10*:mCherry] fish expressing mCherry exclusively in hepatocytes were generated using MultiSite Gateway™ kit (Thermo Fisher Scientific, Waltham, MA, USA) to produce vectors with Tol2 transposon sites^45^. A 2.8-kb promoter of the *fabp10* gene^23^ was cloned into the p5E-mcs vector. Multisite Gateway cloning^46^ was performed with the destination vector pDestTol2pA2, the 5′ entry vector containing the fabp10 promoter, the middle entry vector containing pME-mCherry, and the 3′ entry vector containing p3E-polyA. DNA constructs (25 pg) and Tol2 mRNA (25 pg) were injected into wild-type zebrafish embryos at the one-cell stage.

### Western blotting

Western blotting was performed with antibodies against Lars (#13868; Cell Signaling Technology, Beverly, MA, USA), p62 (PM045; Medical & Biological Laboratories, Nagoya, Japan), LC3B (PM036; Medical & Biological Laboratories), ATG5 (NB110-53818; Novus Biologicals, Littleton, CO, USA), β-actin (A3854; Sigma-Aldrich, St. Louis, MO, USA), and glyceraldehyde 3-phosphate dehydrogenase (GAPDH) (G9295; Sigma-Aldrich). Samples for western blotting were lysed with lysis buffer (0.5% NP-40, 10% glycerin, 50 mM HEPES–KOH (pH 7.8), 150 mM NaCl, and 1 mM EDTA) with protease and phosphatase inhibitor cocktail (Thermo Fisher Scientific). Total proteins were separated by SDS-PAGE, transferred to Immobilon-P membranes (Millipore, Billerica, MA, USA), and probed with the above-mentioned antibodies. Densitometric analysis was performed using Fusion CAPT Advance software version 17.02 (Vilber Lourmat, Collegien, France; https://www.vilber.com/fusion-fx/).

### Reverse-transcription quantitative polymerase chain reaction (RT-qPCR)

The expression of *lars*-related protein genes was analysed using a reverse-transcription quantitative polymerase chain reaction (RT-qPCR). Total RNA was isolated from larvae at 6 dpf using the RNAiso Plus reagent (Takara, Otsu, Japan), as per the manufacturer’s protocol. First-strand cDNA was generated from 0.2 μg RNA using the ReverTra Ace qPCR RT Master Mix with gDNA Remover (Toyobo, Osaka, Japan). After reverse transcription, RT-qPCR was performed using the FastStart Universal SYBR Green Master kit (Roche, Mannheim, Germany) on a Light-Cycler 96 (Roche), according to the manufacturer’s protocol. The following primers were used for zebrafish RT-qPCR: *larsa* and *larsb* (forward), 5′-CAGACAGGAGAGGGAGTTGG-3′; *larsb* (reverse), 5′-GCAGGGCATAAATGGTCTTG-3′; *larsa* (reverse), 5′-TGCAGCTGAAGCATTTAGGA-3′; *lars2* (forward), 5′-CCCGTCACACTGCCTAAAAT-3′; *lars2* (reverse), 5′-GAACCAGCAGCTTCCTGAAC-3′; β-actin (forward), 5′-CGAGCTGTCTTCCCATCCA-3′; β-actin (reverse), 5′-TCACCAACGTAGCTGTCTTTCTG-3′.

### O-dianisidine staining

The embryos at 72 hpf were incubated in o-dianisidine staining buffer (0.6 mg/mL o-dianisidine, 10 mM sodium acetate, 0.65% hydrogen peroxide, and 40% ethanol) for 15 min in the dark.

### Morphological analyses

Zebrafish larvae were placed in 3% methylcellulose, and images were acquired using a Leica M205 FA fluorescent stereo microscope. The liver size was measured manually using ImageJ software (1.52a) (Bethesda, MD, USA; https://imagej.nih.gov/ij/). For the microcephaly assay, the total body length and head diameter through the rear third of the eye lens ratio was measured with LAS X (Leica) and calculated as an index of microcephaly^47,48^.

### Zebrafish survival analysis

Embryos were generated and housed at 28.5 °C. Larvae were transferred to rotifer feeding solution at 5 dpf, and the solution was replaced daily with additional rotifer feeding solution. The dishes were monitored twice a day until 12 dpf.

### Zebrafish locomotion analysis

Locomotion was recorded and analysed as described in Yatsuka et al.^47^. The trajectory plot data were recorded with a Visualix STD1 digital camera (Visualix, Kobe, Japan) attached to a Leica M80 microscope. The trajectory plot data were analysed using SMART video tracking software version 3.0.06 (PanLab, Harvard Apparatus, MA, USA; https://www.panlab.com/en/products/smart-video-tracking-software-panlab). Larvae at 6 dpf were placed into 12-well plates (one larva/well) in 1000 μl embryo medium (0.03% saltwater). The plate was placed under the Leica M80 microscope and tracked as follows: 30 min adaptation and 10 min tracking. All data of the locomotion analysis were recorded and analysed with the Zantiks MWP (Zantiks, Cambridge, UK). Larvae at 6 dpf were placed into 12-well plates (one larva/well) in 1000 μl embryo medium. The plate was transferred to the Zantiks MWP and tracked as follows: 30 min adaptation and 10 min tracking.

### Histopathological staining and fluorescent immunostaining

Small larvae specimens were fixed with 0.1% glutaraldehyde in 4% paraformaldehyde for approximately 48 h, and washed with phosphate-buffered saline. Then, the specimens were washed with gradually increasing concentrations of dimethylformamide and embedded in LR White resin (London Resin Company, Berkshire, UK). Histological examinations were performed using semi-thin sections (1 µm thick) and stained with toluidine blue dye. A double-labelling immunofluorescence analysis was performed on the semi-thin sections using the following primary antibody: rabbit polyclonal LC3B antibody (ab51520; Abcam, Cambridge, UK; 1:100). The secondary antibody used was Alexa Fluor 488 goat anti-rabbit IgG (A31627; Molecular Probes, Eugene, OR, USA; 1:500). Vectashield DAPI (H-1200-10; Vector Laboratories, Brussels, Belgium) was used as a nuclear marker. A laser scanning confocal microscope (BZ-X800, Keyence, Osaka, Japan) equipped with a × 100 oil immersion objective was used to visualize immunoreactivity.

### Immunoelectron microscopy

The ultrastructural localization of LC3B was examined using zebrafish larvae, employing the post-embedding method as described previously^49,50^. Small larvae specimens embedded in LR White Resin, prepared as semi-thin sections, were used. The RFP antibody was used for the detection of mCherry protein, because it reacts with RFP and other RFP variants, such as mCherry. Ultra-thin sections (70 nm thick) were cut, incubated with a rabbit polyclonal LC3B antibody (1:300) and a mouse monoclonal RFP antibody (1:100) for 2 h at 24 °C , and reacted with 10-nm gold colloidal particle-conjugated anti-rabbit IgG (EMGFAR10; British BioCell International, Cardiff, UK; 1:30) and 5-nm gold colloidal particle-conjugated anti-mouse IgG (EMGMHL5; British BioCell International; 1:30). Finally, the sections were stained with lead citrate and examined using a JEM-1400 electron microscope at 80 kV (JEOL, Tokyo, Japan).

### Morpholino oligonucleotide injection

Morpholino oligonucleotide for *atg5* (5′-CATCCTTGTCATCTGCCATTATCAT-3′) was obtained from Gene-Tools, LLC (Philomath, OR, USA). The *atg5* morpholino oligo was used to inhibit *atg5* translation by binding to *atg5* initiation sites^29^. *Atg5* morpholino oligo or control morpholino oligo (0.02 pmol) was injected into the zebrafish eggs at the one-cell stage.

### Bafilomycin A1, chloroquine, 3-methyladenine, and rapamycin treatments

Embryos were treated with bafilomycin A1 (2.5 nM; EMD Millipore, Darmstadt, Germany), chloroquine (10 nM; Sigma-Aldrich), 3-methyladenine (5 mM; Sigma-Aldrich), rapamycin (5 μM; LC Laboratories, Woburn, MA, USA), or dimethyl sulfoxide (DMSO) as the control, in embryo medium from 48 to 72 hpf for morphological experiments and in the larval stage from 4 to 13 dpf for survival experiments. Embryos were treated with rapamycin (5 μM; LC Laboratories, Woburn, MA, USA) or DMSO in embryo medium from 60 to 96 hpf for the morphological experiments^17^ and in the larval stage from 4 to 13 dpf for the survival experiments. The water containing the drug was replaced daily.

### Statistics

Statistical analyses were performed using GraphPad Prism software version 8 (GraphPad Software, Inc., San Diego, CA, USA; https://www.graphpad.com/scientific-software/prism/). All values are expressed as mean ± SEM. Comparisons between groups were made by Student’s t-test. Statistical difference for survival curves were analysed using a Log-rank (Mantel-Cox) test. P < 0.05 was considered statistically significant.

## Supplementary Information

Supplementary Figures.
