# Supplementary material for: Leucyl-tRNA synthetase deficiency systemically induces excessive autophagy in zebrafish
Source: Sci Rep. 2021 Apr 16;11:8392. doi: 10.1038/s41598-021-87879-4 (PMC8052342; doi:10.1038/s41598-021-87879-4)
Supplement: Supplementary file 1 — Supplementary Figures. [file 41598_2021_87879_MOESM1_ESM.pdf]

*Supplementary Information*

# Leucyl-tRNA synthetase deficiency systemically induces excessive autophagy in zebrafish

Masanori Inoue[a, b], Hiroaki Miyahara[c], Hiroshi Shiraishi[a], Nobuyuki Shimizu[a], Mika Tsumori[b], Kyoko Kiyota[b], Miwako Maeda[b], Tohru Ishitani[d], Ryohei Umeda[e], Reiko Hanada[e], Kenji Ihara[b,*] & Toshikatsu Hanada[a,*]

[a] Department of Cell Biology, Oita University Faculty of Medicine, Yufu, Oita 879-5593, Japan

[b] Department of Pediatrics, Oita University Faculty of Medicine, Yufu, Oita 879-5593, Japan

[c] Department of Neuropathology, Institute for Medical Science of Aging, Aichi Medical University, Aichi 480-1195, Japan

[d] Department of Homeostatic Regulation, Division of Cellular and Molecular Biology, Research Institute for Microbial Diseases, Osaka University, Osaka 565-0871, Japan

[e] Department of Neurophysiology, Oita University Faculty of Medicine, Yufu, Oita 879-5593, Japan

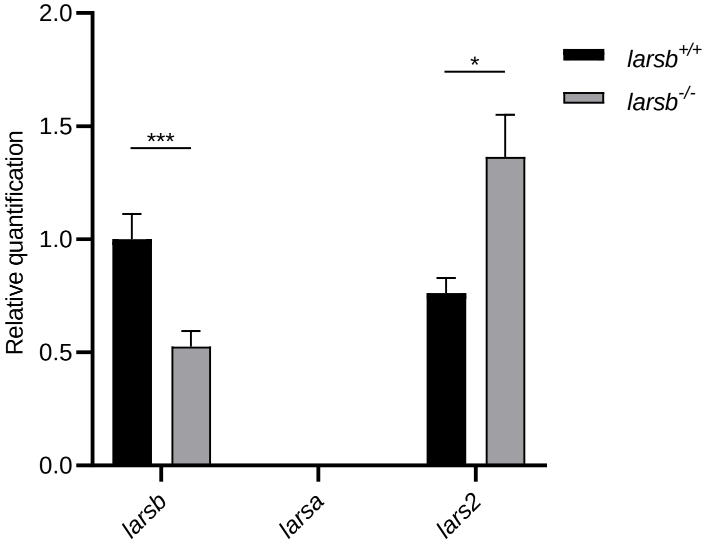


**Supplementary Figure S1.** Quantitative PCR of *larsa*, *larsb*, and *lars2* mRNAs in *larsb*$^{+/+}$ and *larsb*$^{-/-}$ larvae at 6 dpf. n = 6 sample/group. Each sample contains 4 larvae. Error bars indicate SEM. Student's t-test; *P < 0.05, ***P < 0.001. Statistics were calculated and the figure was produced in GraphPad software version 8 (https://www.graphpad.com/scientific-software/prism/)

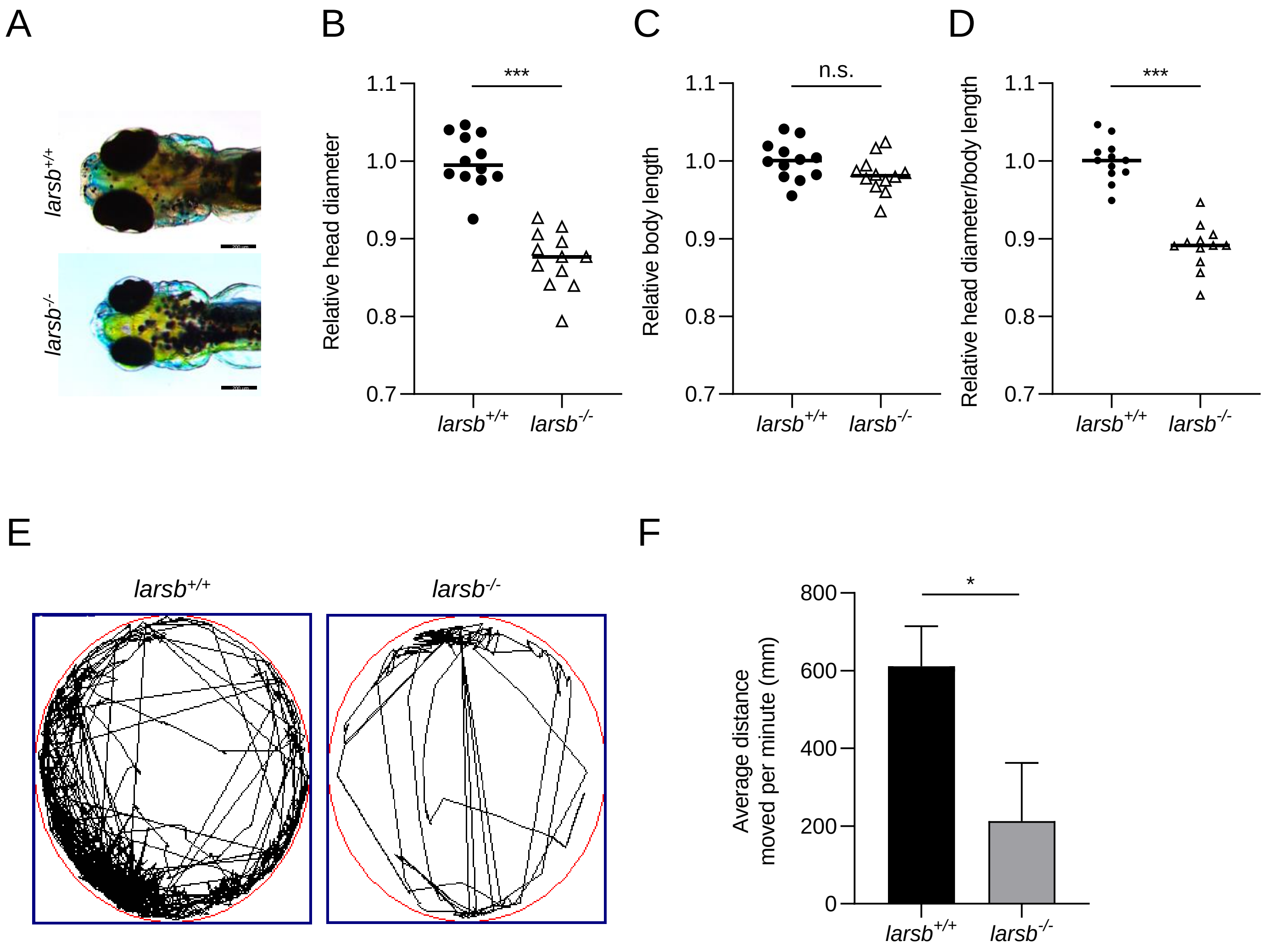


**Supplementary Figure S2.** (A) Brightfield dorsal views of the heads in *larsb*$^{+/+}$ and *larsb*$^{-/-}$ larvae at 6 dpf. Scale bars: 200 µm. (B) Relative head diameter in *larsb*$^{+/+}$ and *larsb*$^{-/-}$ larvae at 6 dpf. n = 12 fish/group. ***p < 0.001. (C) Relative body length in *larsb*$^{+/+}$ and *larsb*$^{-/-}$ larvae at 6 dpf. n = 12 fish/group. n.s.: not significant. (D) Relative head diameter/body length in *larsb*$^{+/+}$ and *larsb*$^{-/-}$ larvae at 6 dpf. n = 12 fish/group. **p < 0.01. (E) Representative trajectory plot of *larsb*$^{+/+}$ and *larsb*$^{-/-}$ larvae at 6 dpf tracked for 10 min. The trajectory plot data were analysed using SMART video tracking software version 3.0.06 (https://www.panlab.com/en/products/smart-video-tracking-software-panlab). (F) Locomotor activity of *larsb*$^{+/+}$ (n = 11) and *larsb*$^{-/-}$ (n = 10) larvae at 6 dpf. Error bars indicate SEM. *p < 0.05. Statistics were calculated and the figure was produced in GraphPad software version 8 (https://www.graphpad.com/scientific-software/prism/)

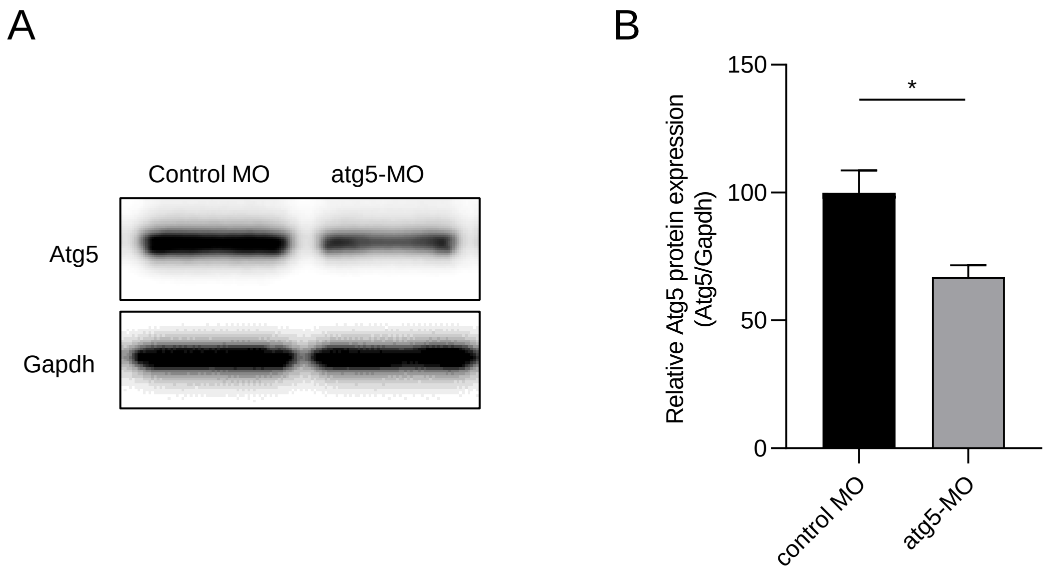


**Supplementary Figure S3.** (A) Western blot analysis for Atg5 protein in wild-type embryos injected with control MO or atg5-MO at 72 hpf. Gapdh levels served as the loading control**.** (B) Densitometric quantification of the relative ratio of Atg5 protein to Gapdh protein in three independent experiments. Densitometric analysis was performed using Fusion CAPT Advance software version 17.02 (https://www.vilber.com/fusion-fx/). Error bars indicate SEM. Student's t-test; *P < 0.05. Statistics were calculated and the figure was produced in GraphPad software version 8 (https://www.graphpad.com/scientific-software/prism/) MO: morpholino; Gapdh: Glyceraldehyde 3-phosphate dehydrogenase.

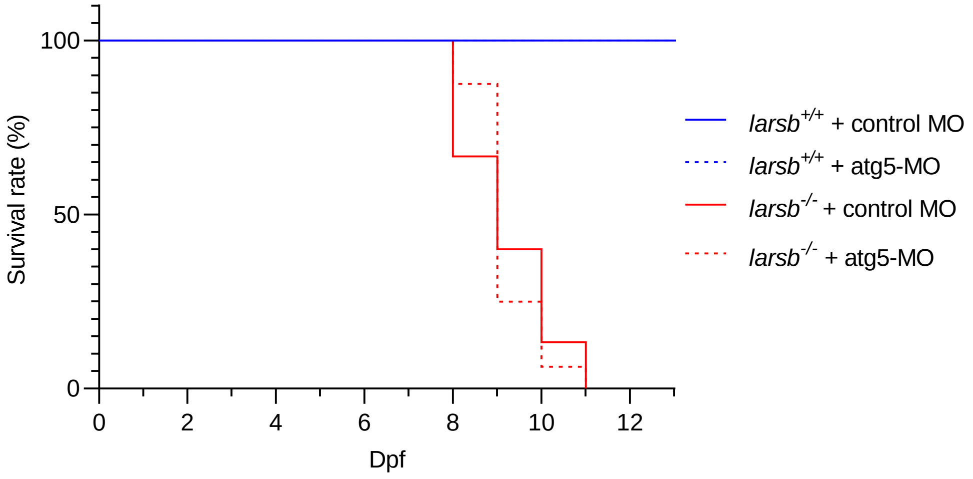


**Supplementary Figure S4.** Kaplan–Meier survival curve of *larsb*$^{+/+}$ (n = 22) and *larsb*$^{-/-}$ (n = 15) injected with control MO, and *larsb*$^{+/+}$ (n = 22) and *larsb*$^{-/-}$ (n = 16) injected with atg5-MO. Statistics were calculated and the figure was produced in GraphPad software version 8 (https://www.graphpad.com/scientific-software/prism/) Lars: leucyl-tRNA synthetase; MO: morpholino; Dpf: days post fertilization.

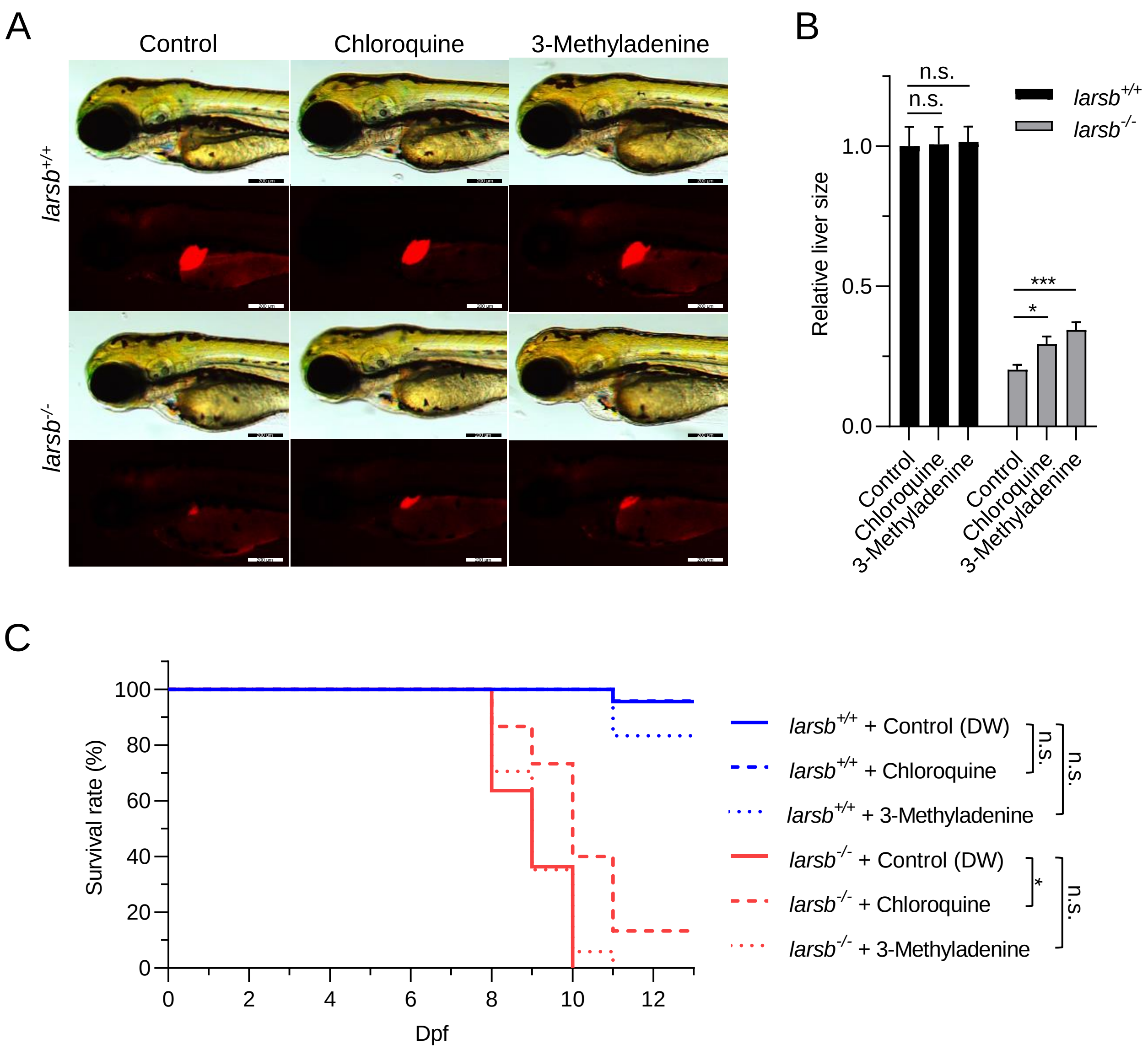


**Supplementary Figure S5.** (A) Morphological abnormality at 72 hpf in the livers of *larsb*$^{-/-}$ larvae under Tg[*fabp10*:mcherry] background treated with control (DW), chloroquine and 3-methyladenine. Scale bars: 200 μm. (B) Quantification of liver size in *larsb*$^{-/-}$ larvae under Tg[*fabp10*:mcherry] background (72 hpf). Liver sizes were evaluated using ImageJ software (1.52a) (https://imagej.nih.gov/ij/). n = 10 fish/group. Error bars indicate SEM. Student's t-test; *P < 0.05, ***P < 0.001. (C) Kaplan–Meier survival curve of *larsb*$^{+/+}$ (n = 23) and *larsb*$^{-/-}$ (n = 11) larvae treated with control (DW), *larsb*$^{+/+}$ (n = 24) and *larsb*$^{-/-}$ larvae (n = 15) treated with chloroquine and *larsb*$^{+/+}$ (n = 24) and *larsb*$^{-/-}$ larvae (n = 17) treated with 3-methyladenine. Statistics were calculated and the figure was produced in GraphPad software version 8 (https://www.graphpad.com/scientific-software/prism/) Lars: leucyl-tRNA synthetase; n.s.: non-significant; DW: distilled water; Dpf: days post fertilization.

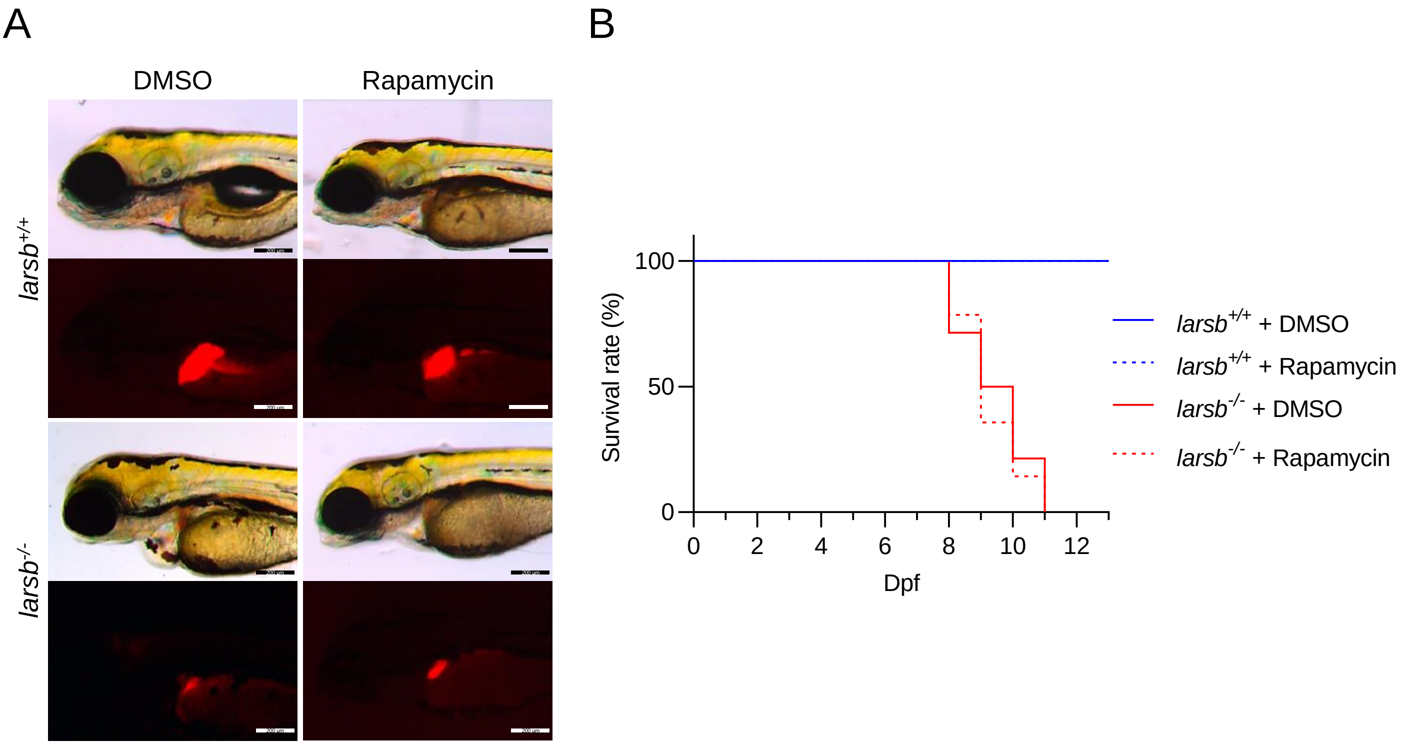


**Supplementary Figure S6. (A)** Morphological abnormality at 96 hpf in the livers of *larsb*$^{-/-}$ larvae under Tg[fabp10:mcherry] background treated with DMSO or rapamycin. Scale bars: 200 µm. (**B)** Kaplan–Meier survival curve of *larsb*$^{+/+}$ (n = 33) and *larsb*$^{-/-}$ (n = 14) treated with DMSO, and *larsb*$^{+/+}$ (n = 40) and *larsb*$^{-/-}$ (n = 14) treated with rapamycin. Statistics were calculated and the figure was produced in GraphPad software version 8 (https://www.graphpad.com/scientific-software/prism/) Lars: leucyl-tRNA synthetase; DMSO: dimethyl sulfoxide; Dpf: days post fertilization.

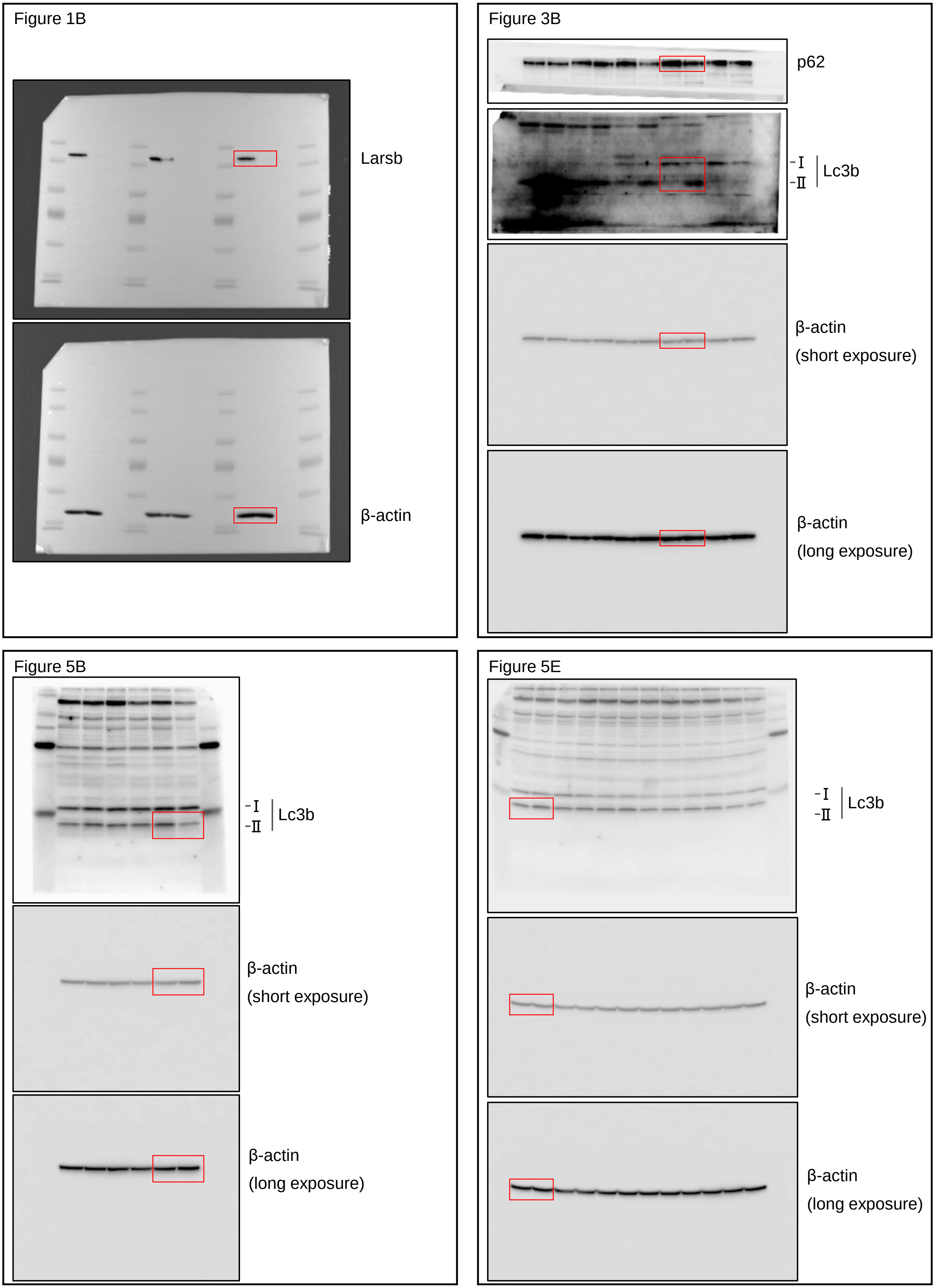


**Supplementary Figure S7.** Uncropped Western blots, shown in Figure 1B, 3B, 5B and 5E. Cropped areas are highlighted by red box.

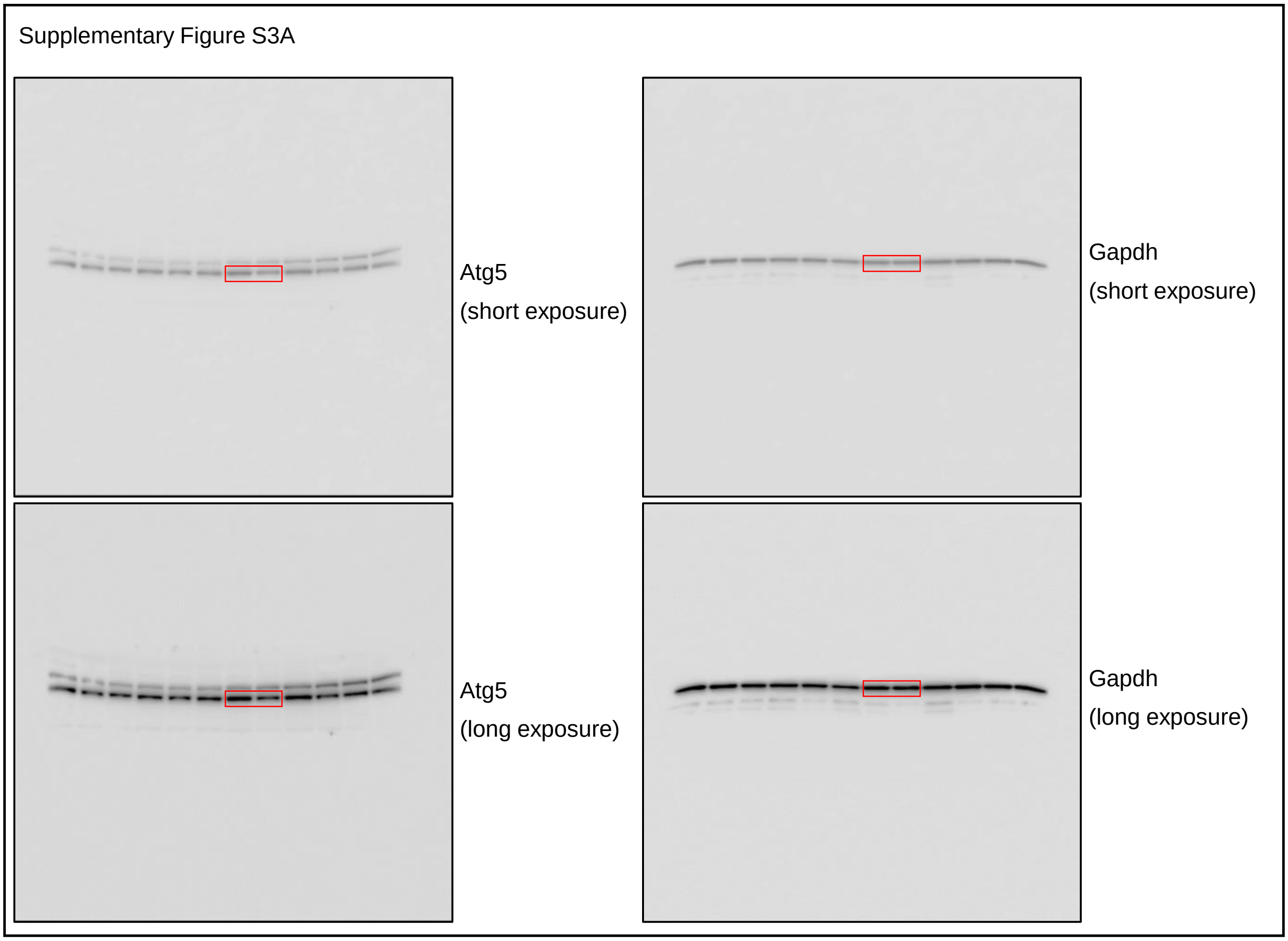


**Supplementary Figure S8.** Uncropped Western blots, shown in Supplementary Figure S3A. Cropped areas are highlighted by red box.
